# Investigating the association between cancer and dementia risk: a longitudinal cohort study

**DOI:** 10.1186/s13195-022-01090-9

**Published:** 2022-10-05

**Authors:** Dan-Dan Zhang, Ya-Nan Ou, Liu Yang, Ya-Hui Ma, Lan Tan, Jian-Feng Feng, Wei Cheng, Jin-Tai Yu

**Affiliations:** 1 Department of Neurology, Qingdao Municipal Hospital, Qingdao University, Qingdao, China; 2 Department of Neurology and Institute of Neurology, Huashan Hospital, State Key Laboratory of Medical Neurobiology and MOE Frontiers Center for Brain Science, Shanghai Medical College, Fudan University, 12th WulumuqiZhong Road, Shanghai, 200040 China; 3 Institute of Science and Technology for Brain-inspired Intelligence, Fudan University, Shanghai, China

**Keywords:** Alzheimer’s disease, Dementia, Vascular dementia, Cancer, Epidemiology

## Abstract

**Background:**

Previous studies found that cancer survivors had a reduced risk of dementia compared with the general population. However, these findings were uncertain because of survivor bias and a lack of stratification by cancer types. This current cohort study used data from the UK Biobank to explore these associations.

**Methods:**

Multivariable Cox regression analyses were used to examine the association of cancer status and the risk of dementia with its subtypes after adjusting for age and sex. Hazard ratios (HRs) with 95% confidence intervals (CIs) were calculated as a measure of relative risk by comparing observed dementia incidence among cancer patients.

**Results:**

We included 263,151 participants in the observational analysis. During a median follow-up of 9.18 years, dementia was diagnosed in 472 individuals with cancer and 3685 individuals without cancer, respectively. Cancer patients had lower risks of dementia (hazard ratio: 0.89, confidence interval: 0.81–0.98) and its subtypes (Alzheimer’s disease [AD]: 0.85 [0.74–0.98]; vascular dementia [VD]: 0.81 [0.66–0.99]) in the Cox regression adjusted for age and sex. Individuals with cancers in the male genital system had substantially reduced risks of dementia (0.66 [0.46–0.93]) and AD (0.53 [0.29–0.97]) than those with cancers in other systems. Moreover, non-melanoma skin cancer and prostate cancer were associated with a reduced risk of dementia (0.79 [0.62–0.99]; 0.69 [0.49–0.97]), but not with AD or VD (*P*>0.05).

**Conclusions:**

The current study supported a negative association between cancer and dementia risk, and encourages further exploration of the mechanistic basis of this inverse relationship to improve understanding.

**Supplementary Information:**

The online version contains supplementary material available at 10.1186/s13195-022-01090-9.

## Background

With the aging population trend, dementia and cancer which were common in the elderly population have become major public health problems, placing a substantial burden on patients, their families, and the national healthcare systems [1, 2]. The incidence and prevalence of age-related diseases including cancer and dementia are rapidly increasing in recent years. Cancer is a major public health concern worldwide and the second leading cause of death in the USA [3]. In both sexes combined, lung cancer is the most commonly diagnosed cancer, closely followed by female breast cancer, prostate cancer, colorectal cancer, and it is also the leading cause of cancer death, followed by colorectal cancer, stomach cancer, and liver cancer [4]. Improvements in early diagnosis and treatment have ensured longer survival of cancer patients, which in turn increase the incidences of the long-term side effects such as dementia. Dementia is characterized by a constant decline and deterioration of mental capacity, which inevitably impairs quality of life [5]. Generally, dementia refers to all-cause of dementia (ACD), of which Alzheimer’s disease (AD) and vascular dementia (VD) are its 2 main subtypes. Dementia prevention is of the high priority given the limited therapeutic value of drugs currently used to treat dementia [6].

Several epidemiological studies have shown reductions in risk for dementia and AD subsequent to a cancer diagnosis [7–12]. A possible explanation for the inverse relationship was that dementia and cancer had opposing pathological processes, i.e., uncontrolled cell proliferation in malignancy and neuronal cell death in dementia and AD [13–16]. An alternative explanation for this negative association is that these studies failed to take into consideration the competing risk of death in cancer patients, whose mortality rates were higher compared to individuals without cancer [17]. A recent cohort study supported this protective effect of cancer on subsequent dementia diagnosis (hazard ratio [HR]: 0.58, 95% confidence interval [CI]: 0.35–0.97) and AD diagnosis (HR: 0.45, 95%CI: [0.24–0.85]) [18]. The investigators explored the relationship between incident cancer and dementia by using illness-death models to account for survivor bias. But they cannot investigate differential effects on dementia risk according to cancer type because of the limited cases [18]. Furthermore, other studies reported similar findings but also had parallel limitations [7, 8, 10, 12, 19, 20]. These limitations included relatively short follow-up periods [7, 8, 19], small sample sizes [7, 11, 18], or a lack of information about relevant comorbidities [10, 19].

In this national cohort study, we comprehensively explored the risk for ACD, AD, and VD among patients with a cancer diagnosis compared to risk within the UK white adult population. Then we further assessed the organ specificity of the associations by mapping each cancer with the risk of dementia. And we also assessed the effect of potentially confounding factors on dementia risk among cancer patients.

## Methods

This prospective cohort study was based on data from the UK Biobank study [21] that received approval from the National Information Governance Board for Health and Social Care and the National Health Service North West Multicenter Research Ethics Committee. All participants provided informed consent through electronic signature at baseline assessment.

### Study population

The UK Biobank was a population-based cohort of more than 500,000 participants who attended 1 of 22 assessment centers across the United Kingdom (UK) between 2006 and 2010 [21]. The International Statistical Classification of Diseases (ICD) coding system was used to record disease diagnoses. Among 502,486 participants with available data in the cancer register, non-malignant neoplasms were not included in the definition of cancer, and non-white people were excluded. Then, individuals younger than 50 years old at baseline and those younger than 40 years old at the time of cancer diagnosis were excluded, and individuals with prevalent dementia at baseline were also excluded. Furthermore, among the remaining 319,923 eligible participants, individuals without definite dementia information diagnosis, those with cancer diagnosed after enrollment, and those with a cancer diagnosis within one year before the dementia diagnosis were excluded. The diagnosis date was the date when the ICD-10 code was first recorded in participants’ inpatient records. After applying the exclusion criteria, 263,151 participants were included in the analysis.

### Ascertainment of cancer cases

We identified malignant tumors and compared dementia risk between individuals with and without a cancer history. In this study, cancer was ascertained via linkage to cancer registry and death certificate data. National cancer registries centralize information received from separate regional cancer centers around the UK. For analyses of individual cancer types and systems, we focused on the 18 specific cancers and 6 major systems with at least 100 affected individuals and at least 10 dementia cases among the affected individuals. Then we focused on the 5 cancer types with at least 1000 affected individuals as common cancers. Cancer cases within UK Biobank were identified by ICD-10 codes through linkage to the national cancer registry (C01-C97, D00-D48). To avoid potential survivor bias associated with incident cancer cases, this study was restricted to prevalent cancer cases.

### Ascertainment of dementia cases

Clinical outcomes including ACD, AD, and VD diagnoses were available over a follow-up period extending up to 2021. Dementia was ascertained using hospital in-patient records containing data on admissions and diagnoses obtained from the Hospital Episode Statistics for England, Scottish Morbidity Record data for Scotland, and the Patient Episode Database for Wales. Additional cases were detected through linkage to death register data provided by the National Health Service Digital for England and Wales and the Information Services Division for Scotland. Diagnoses were recorded using the ICD-10 coding system. For the current analyses, the algorithmically defined ACD outcomes (Fields 42,018 and 42,019), AD outcomes (Fields 42,020 and 42,021), and VD outcomes (Fields 42,022 and 42,023) were used [22]. Participants with dementia were identified as having a primary/secondary diagnosis (hospital records) or underlying/contributory cause of death (death register) using ICD-9 and ICD-10 codes for AD and other dementia classifications [23]. A recent study suggested that dementia diagnoses can be reliably identified from primary care, hospital admissions, and mortality data with a positive predictive value (PPV) of 82.5% for ACD combining all data sources. PPVs for all datasets combined were 71.4% and 43.8% for AD and VD, respectively [24].

### Assessment of covariates

We considered potential confounders to be any variable suspected to be linked to dementia incidence. All main models were adjusted for age at recruitment (Field 21,022) and sex (Field 31). For the extended model, we considered the following additional variables: education, categorized as higher (college/university degree or other professional qualification) or lower (Field 6138); socioeconomic status, categorized as quintiles 1, 2 to 4, and 5 (Field 189: Townsend deprivation index [combining information on social class, employment, car availability and housing]); apolipoprotein E (*ApoE*) ε4 carrier status (carrier/non-carrier status as defined by genetic information); body mass index (BMI, Field 21,001); smoking status (Field 20,116); alcohol consumption (Field 100,580); history of diabetes (Filed 130,706–130,714); history of hypertension (Field 131,286); obesity (Field 130,792); history of heart failure (Field 131,354); and history of stroke (Field 131,368).

In addition, we adjusted for cognitive function as an additional confounder, as medical treatments for the cancer were found to cause a decline in the memory [25]. In the current study, we focused on the two tests available to the greatest number of participants (pairing and reaction time) to reflect cognitive function. Pair matching (PM, Field 399) and reaction time (RT, Field 20,023) measure visual memory and reaction ability, respectively, with higher scores reflecting worse cognitive function (continuous scores).

### Statistical analysis

All participants were followed up from the date of recruitment until that of dementia diagnosis, death, loss to follow-up, or the end of the study period (November 6, 2021), whichever occurred first. Individuals with prevalent cancer reported at baseline enrollment were classified as the exposure group. Baseline characteristics of the analytic sample were summarized across cancer status as a percentage for categorical variables and mean and standard deviation (SD) for continuous variables.

Multivariable Cox regression analyses were used to examine the associations of cancer status with incident ACD, AD, and VD. Hazard ratios (HRs) with 95% confidence intervals (CIs) were calculated as a measure of relative risk by comparing the observed dementia incidence among cancer patients with that of non-cancer participants. Two multivariable models were adjusted for different variables. Model 1 adjusted for age and sex. To examine whether the association of dementia with cancer was attributable to lifestyle factors and other health-related factors, model 2 was additionally adjusted for education, socioeconomic status, *ApoE4*, BMI, smoking status, alcohol consumption, obesity, hypertension, diabetes, heart failure, and stroke.

We conducted several sensitivity analyses. To explore the effect of cognitive function on the incidence of dementia in participants, we additionally adjusted for cognitive function (PM and RT). To determine the potential differential effect of cancer on dementia type, three definitions of the outcome were compared: (1) all-cause dementia; (2) AD dementia; and (3) vascular dementia. The cumulative incidence curve for each cohort was measured using the Kaplan–Meier method, and the curve difference was calculated using the log-rank test. Since cancer is a significant risk factor for death, it was necessary to test the possible influence of competing events on the association between cancer and the risk of dementia by applying Fine and Gray’s sub-distribution hazards regression model [26]. The competing event was defined as death. We also performed subgroup analyses stratified by baseline characteristics of the participants. In addition, we conducted separate subgroup analyses according to whether participants had non-melanoma skin cancer [NMSC] or other cancers except NMSC, whether the cancer is related to smoking-related cancers (oral, pharynx, larynx, esophagus, stomach, pancreas, bronchus or lung, cervix uteri, bladder, and kidney) [27], or not, and whether participants had a cancer in 18 specific organs or a cancer in 6 major systems. *P* values were 2-sided with statistical significance set at less than 0.05. All statistical analyses were performed using R statistical software, version 4.1.1.

## Results

### Characteristics of the study population

**Fig. 1:**
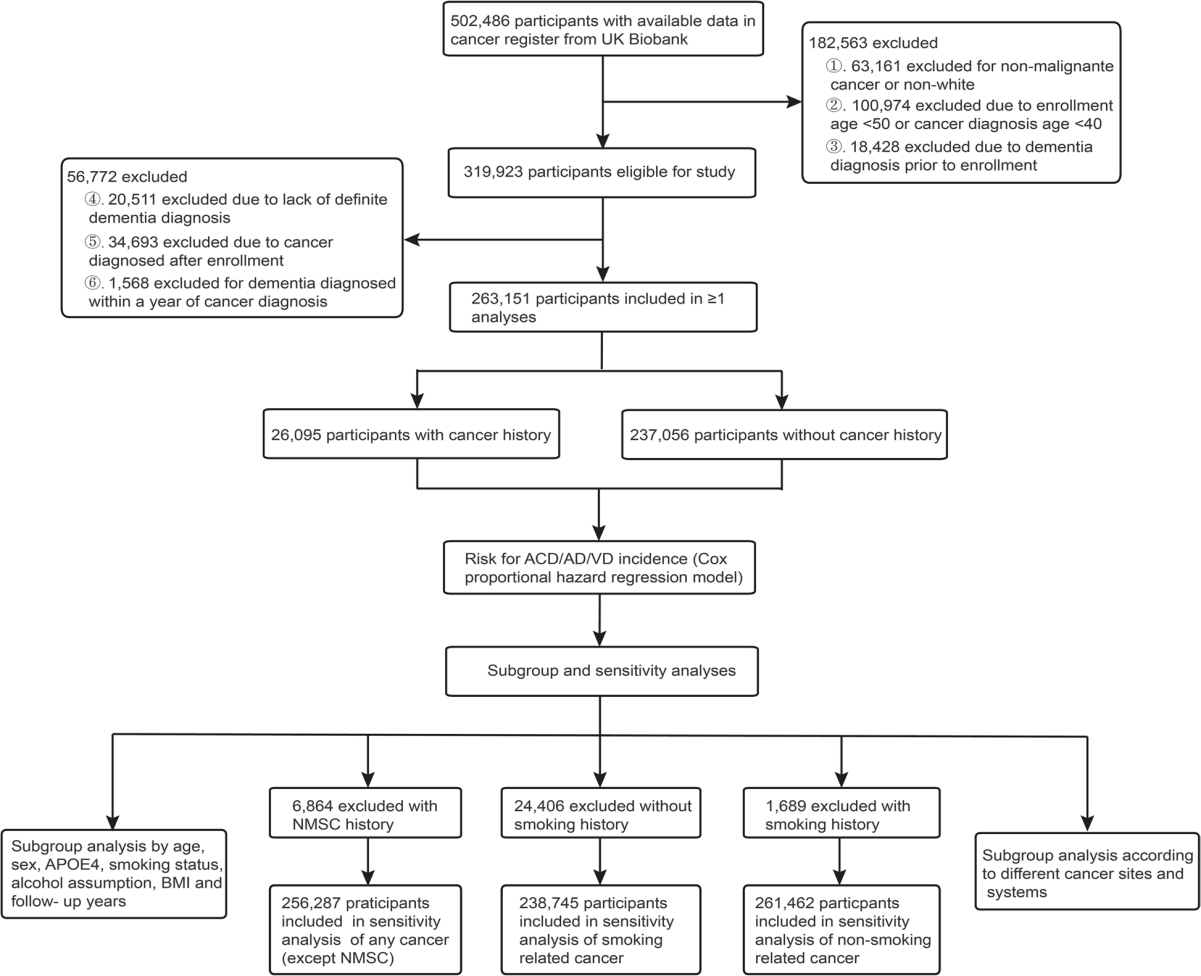
Flowchart for the selection of the analyzed study sample from the UK Biobank study. Abbreviations: ACD, all-cause dementia; AD, Alzheimer’s disease; VD, vascular dementia; *ApoE4*, apolipoprotein E ε4; BMI, body mass index; NMSC, non-melanoma skin cancer

At baseline, 502,486 participants were screened. After applying the exclusion criteria, 263,151 participants were included in our study (Fig. 1). The mean age at recruitment was 60.27 (SD: 5.40) years, and 142,824 participants (54.27%) were female. The median follow-up was 9.28 (interquartile range [IQR]: 5.55–13.01) years for cancer patients and 9.17 (IQR: 5.65–12.69) years for non-cancer patients. Baseline characteristics of participants were shown in Table 1.

**Table 1:** Study characteristics of analytical sample stratified by cancer status

Characteristics	Participants with cancer history (*n*=26,095 )	Participants without cancer history (*n*=237,056 )
Age at cancer diagnosis, mean (SD), years	62.14 (5.02)	60.07 (5.40)
BMI, mean (SD)	27.47 (4.70)	27.68 (4.78)
Age at entry, *n* (%)		
50–60 years	7201 (27.60)	102,718 (43.33)
≥60 years	18,894 (72.40)	134,338 (56.67)
Sex (female, %)	15,348 (58.81)	127,476 (53.77)
*ApoE4* carrier	7088 (27.16)	65,523 (27.64)
College, *n* (%)		
Yes	11,101 (42.54)	103,445 (43.64)
No	8310 (31.85)	78,651 (33.18)
Unknown	6684 (25.61)	54,960 (23.18)
Smoking, *n* (%)		
Never	13,111 (50.24)	124,033 (52.32)
Previous	10,678 (40.92)	89,766 (37.87)
Current	2182 (8.36)	22,317 (9.41)
Unknown	124 (0.48)	940 (0.40)
Alcohol, *n* (%)		
Never	1069 (4.10)	8376 (3.53)
Previous	1026 (3.93)	8704 (3.67)
Current	23,972 (91.86)	219,753 (92.70)
Unknown	28 (0.11)	223 (0.09)
Co-morbidities, *n* (%)		
Diabetes	3026 (11.60)	24,973 (10.53)
Hypertension	11,686 (44.78)	92,207 (38.90)
Stroke	1997 (7.65)	14,857 (6.27)
Obesity	2800 (10.73)	26,007 (10.97)
Heart failure	5922 (22.69)	44,007 (18.56)
Follow-up years, median (interquartile range)	9.28 (5.55-13.01)	9.17 (5.65-12.69)
Types of dementia		
ACD, *n* (%)	472 (1.81)	3685 (1.55)
AD, *n* (%)	208 (0.80)	1670 (0.70)
VD, *n* (%)	103 (0.39)	862 (0.36)

*Abbreviations*: *BMI* body mass index, *ACD* all-cause dementia, *AD* Alzheimer’s disease, *VD* vascular dementia

Model 1 was adjusted for age and sex and model 2 was additionally adjusted for education, socioeconomic status, *ApoE4*, BMI, smoking status, alcohol consumption, obesity, hypertension, diabetes, heart failure, and stroke. After adjustment for demographic characteristics (age, sex, education, socioeconomic status), BMI, smoking status, and alcohol consumption were included in model 2, taking into account the established effects on cancer-related mortality [18]. In addition, *ApoE4* and some comorbidities (obesity, hypertension, diabetes, heart failure, and stroke) were also included in model 2 as risk factors for dementia [8, 28]. The subgroup analyses and sensitivity analyses in the article were based on Model 2.

### Risk of dementia after cancer

**Fig. 2:**
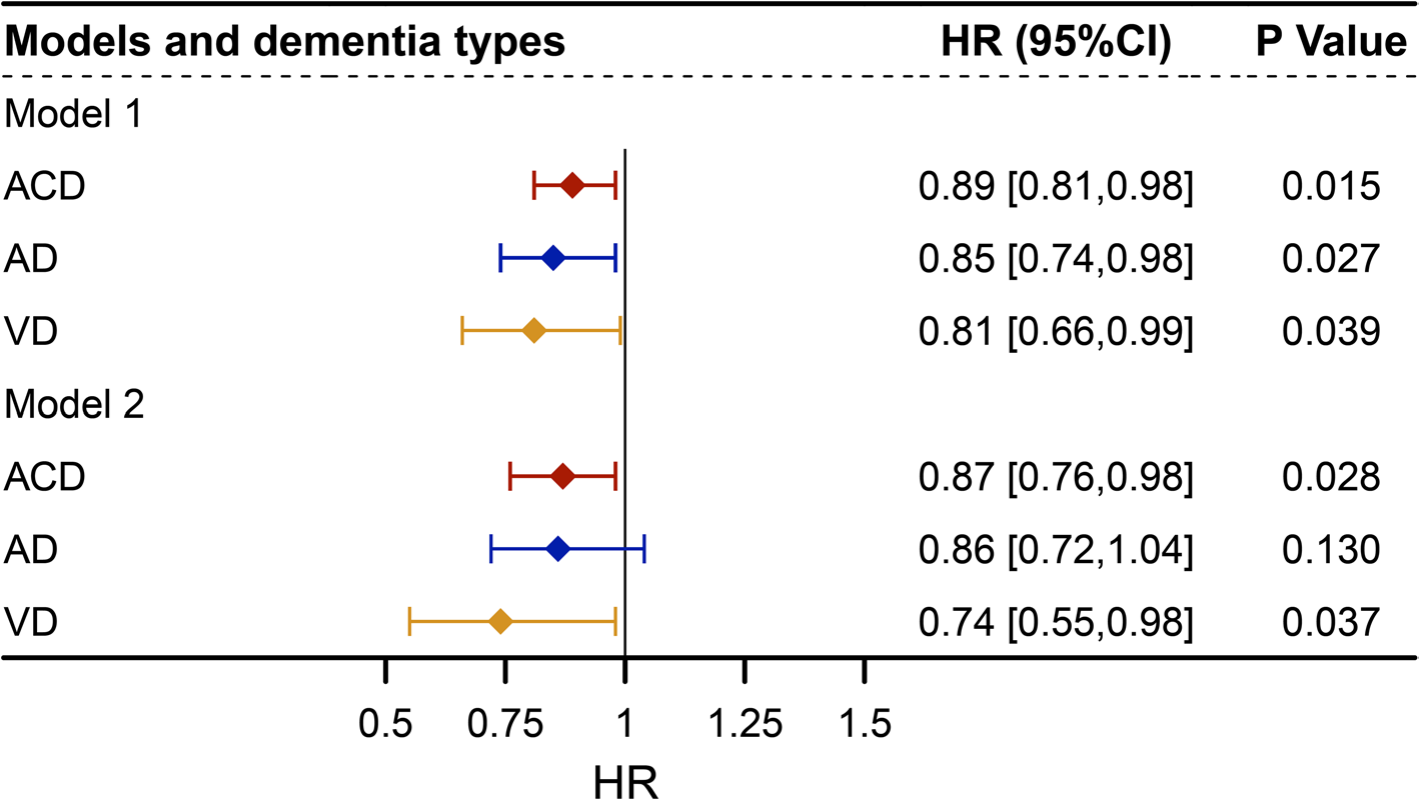
Multi-state results: HRs and 95% CIs of cancer on the transition to dementia. Model 1 adjusted for age at study entry, and sex. Model 2 further adjusted for education, BMI, *ApoE4*, smoking status, alcohol consumption, socioeconomic status, diabetes, hypertension, heart failure, obesity, and stroke. Abbreviations: HR, hazard ratios; CI, confidence interval; ACD, all-cause dementia; AD, Alzheimer’s disease; VD, vascular dementia

We included 26,095 participants with a history of cancer at baseline, among whom 472 participants developed ACD, 208 developed AD, and 103 developed VD during the follow-up (Table 1). We investigated the association of cancer with dementia and its subtypes risks by using Cox regression models (Fig. 2). In model 1, the risk of dementia and its subtypes among patients with a cancer history were lower compared to those without a cancer history. Compared with non-cancer patients, HRs (95% CI) for risks of ACD, AD, and VD among patients with cancer were 0.89 (0.81–0.98), 0.85 (0.74–0.98), and 0.81 (0.66–0.99), respectively. In Cox model 2, the inverse association of cancer with ACD (0.87 [0.76–0.98]) and VD (0.74 [0.55–0.98]) risk were still significant (Fig. 2). After additional adjustments for cognitive function, cancer was only associated with a reduced risk of ACD (0.86 [0.76–0.98]), but not AD or VD (*P*>0.05) (Supplementary Table 1).

**Fig. 3:**
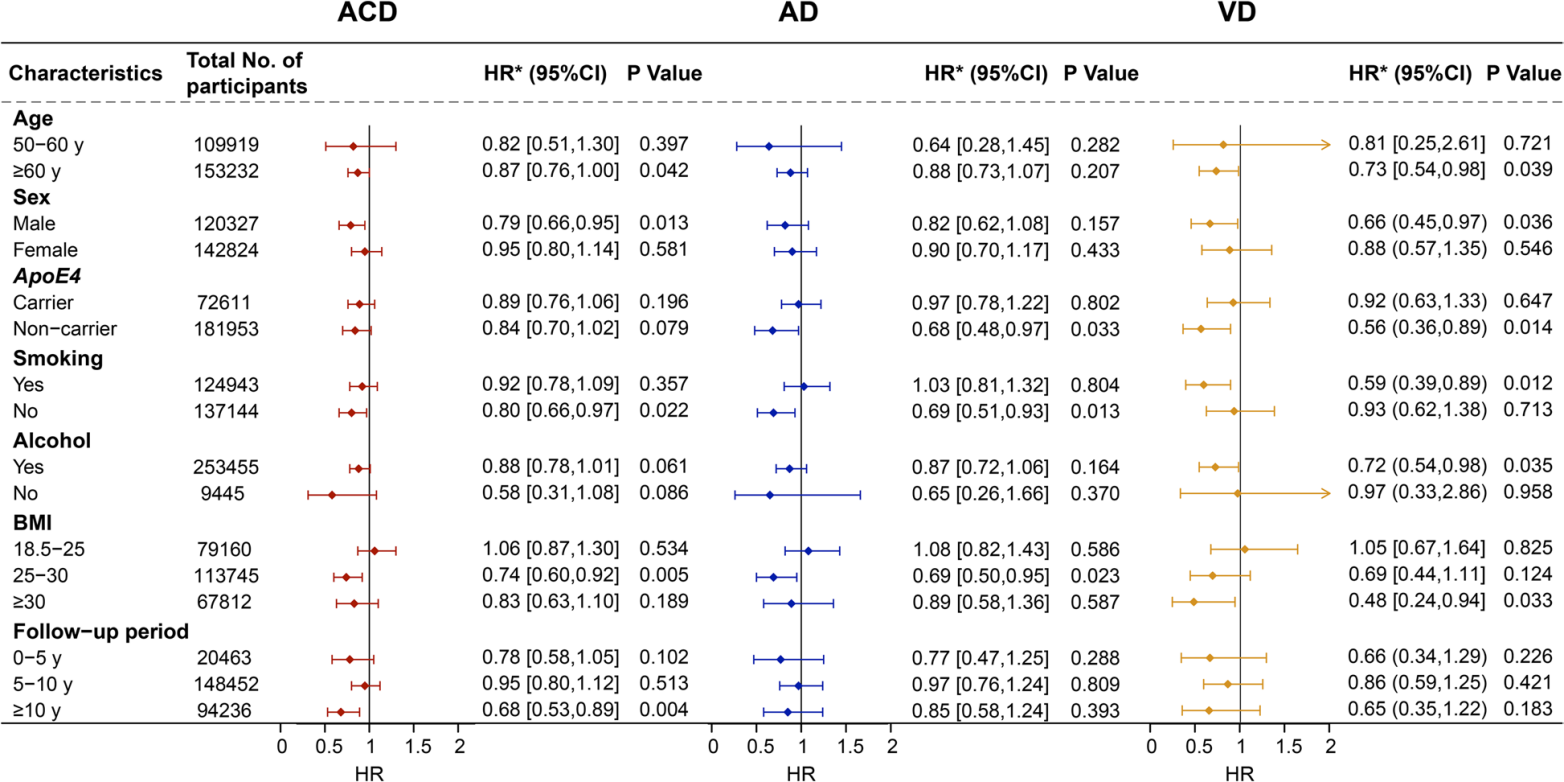
Risk Factors associated with dementia development using the robust inference for the Cox regression. *Calculated using Cox proportional hazards modeling, adjusted for age, sex, education, *ApoE4*, socioeconomic status, BMI, smoking status, alcohol consumption, diabetes, hypertension, heart failure, obesity, and stroke. Abbreviation: ACD, all-cause dementia; AD, Alzheimer’s disease; VD, vascular dementia; HR, hazard ratios; CI, confidence interval

To further investigate the effects of confounding factors on the association between cancer and the risk of dementia, a series of subgroup analyses stratified by age, sex, *ApoE4* status, smoking status, alcohol consumption, BMI [29], and follow-up period were conducted (Fig. 3). These analyses were all adjusted for age, sex, education, socioeconomic status, *ApoE4*, BMI, smoking status, alcohol consumption, obesity, hypertension, diabetes, heart failure, and stroke. The inverse association between cancer and ACD risk was more pronounced in individuals aged over 60 years old (0.87 [0.76–1.00]), males (0.79 [0.66–0.95]), those without smoking history (0.80 [0.66–0.97]), those with a BMI between 25 and 30 (0.74 [0.60–0.92]), or those with a follow-up of more than 10 years (0.68 [0.53–0.89]). A significant inverse association was observed between cancer and AD risk in individuals with *ApoE4* non-carriers (0.68 [0.48–0.97]), individuals without smoking history (0.69 [0.51–0.93]), or those with a BMI between 25 and 30 (0.69 [0.50–0.95]). In addition, the same pattern of associations was also pronounced between cancer and VD risk in individuals aged over 60 years old(0.73 [0.54–0.98]), males (0.66 [0.45–0.97]), *ApoE4* non-carriers (0.56 [0.36–0.89]), those with smoking history (0.59 [0.39–0.89]), those with alcohol history (0.72 [0.54–0.98]), or those with a BMI over 30 (0.48 [0.24–0.94]).

In Kaplan-Meier analysis, the log-rank test showed a difference between the populations being studied in the probability of ACD at any time point, lacking significant differences in the probability of AD and VD (Supplementary Fig. 1). As shown in Supplementary Fig. 2, the cumulative incidences of dementia remained statistically different between cancer and non-cancer groups after controlling for competing risks of death.

### Risk of dementia in different cancer sites and systems

**Fig. 4:**
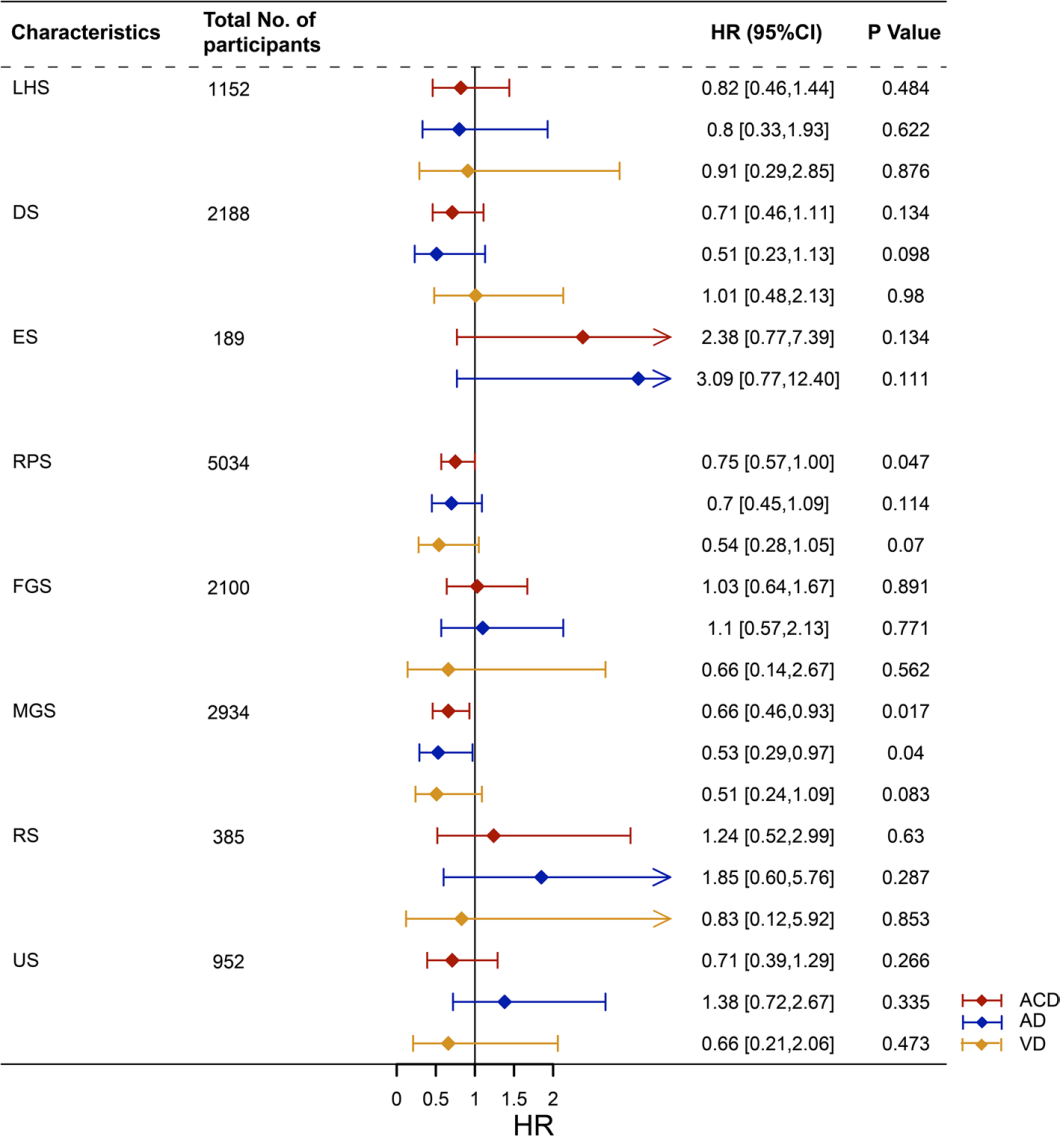
Association of system-specific cancers with dementia and its subtypes. *Calculated using Cox proportional hazards modeling, adjusted for age, sex, education, *ApoE4*, socioeconomic status, BMI, smoking status, alcohol consumption, diabetes, hypertension, heart failure, obesity, and stroke. − The number of people diagnosed with ACD, AD, or VD in the type of cancer patients is too small to calculate the HR value. Abbreviation: ACD, all-cause dementia; AD, Alzheimer’s disease; VD, vascular dementia; HR, hazard ratios; CI, confidence interval; LHS, lymphohematopoietic system; DS, digestive system; ES, endocrine system; RPS, reproductive system; MGS, male genital system; FGS, female genital system; RS, respiratory system; US, urinary system

To further explore the correlation between cancer and dementia risk, we stratified cancer into subgroups of cancers in 6 different systems: lymphohematopoietic system, digestive system, endocrine system, reproductive system (including male genital system and female genital system), respiratory system, and urinary system. We observed that in Cox model 2, only the reproductive system cancer was associated with a lower risk of ACD (0.75 [0.57–1.00]), but not with AD or VD risk (P>0.05). However, cancer patients of the male genital system had substantially reduced risks of ACD (0.66 [0.46–0.93]) and AD (0.53 [0.29–0.97]) (Fig. 4).

In sensitivity analyses, no matter whether it is related to smoking, any cancer (except NMSC) was associated with risk of incident dementia (Supplementary Table 2). Moreover, we assessed the associations of specific cancer sites with dementia risk. In Cox model 2, compared with non-cancer patients, only NMSC and prostate cancer (PC) patients were associated with a lower risk of ACD (NMSC: 0.79 [0.62–0.99]; PC:0.69 [49–0.97]), but not with AD and VD (Supplementary Table 2).

In addition, we stratified our results by age, smoking status, alcohol consumption, *ApoE4* status, and BMI to explore the association between 5 common cancers and dementia risk (Table 2, Supplementary Table 3). In individuals over 60 years old, NMSC and PC patients showed a decreased ACD risk (NMSC: 0.78 [0.61–0.99]; PC: 0.70 [0.49–0.99]), but no observed differences in AD and VD risk. In various subgroups stratified by smoking status, colon cancer patients without a smoking history had an increased risk of VD (3.64 [1.49–8.88]), and NMSC patients with a smoking history had decreased risks of ACD (0.67 [0.48–0.95]) and VD (0.34 [0.13–0.93]). NMSC was associated with VD risk (0.51 [0.27–0.95]) and PC was associated with ACD risk (0.70 [0.50–1.00]) in the drinkers (Table 2). In addition, NMSC was associated with VD risk (0.20 [0.05–0.79]) among patients without *ApoE4*. Melanoma of skin cancer was associated with increased AD (2.77 [1.03–7.45]) and VD (4.48 [1.10–18.32]) risks among patients with BMI between 18 and 25, but NMSC was associated with ACD (0.66 [0.45–0.97]) risk among those with BMI between 25 and 30 (Supplementary Table 3).

**Table 2:** Association of specific cancer sites with dementia by age, smoking status, and alcohol status

Specific cancer sites^a^	Participants with cancer history (N)	ACD			AD			VD		
		HR^b^	95% CI	*P*-value	HR^b^	95% CI	*P*-value	HR^b^	95% CI	*P*-value
Age <60 years										
Breast	2189	0.73	0.27–1.98	0.538	0.41	0.06–2.97	0.378	3.43	0.78–15.03	0.103
Colon	254	-	-	-	-	-	-	-	-	-
Melanoma of skin	350	0.82	0.12–5.87	0.847	1.98	0.28–12.20	0.497			
NMSC	1665	0.97	0.43–2.17	0.936	0.77	0.19–3.12	0.715	-	-	-
Prostate	334	0.67	0.09–4.77	0.686	-	-	-	-	-	-
Age ≥60 years										
Breast	4493	1.04	0.79–1.36	0.787	0.95	0.63–1.41	0.783	0.90	0.46–1.76	0.751
Colon	767	0.89	049–1.62	0.712	0.76	0.29–2.04	0.590	1.73	0.72–4.19	0.223
Melanoma of skin	719	1.14	0.64–2.01	0.655	1.26	0.57–2.82	0.567	0.86	0.21–3.44	0.828
NMSC	5199	**0.78**	**0.61–0.99**	**0.038**	0.79	0.55–1.12	0.184	0.60	0.34–1.06	0.080
Prostate	2378	**0.70**	**0.49–0.99**	**0.044**	0.58	0.32–1.06	0.077	0.56	0.26–1.19	0.130
Smoking										
Breast	2969	1.24	0.86–1.78	0.243	1.18	0.70–1.99	0.538	1.20	0.52–2.74	0.671
Colon	556	0.57	0.21–1.52	0.263	0.35	0.05–2.46	0.289	-	-	-
Melanoma of skin	440	1.51	0.75–3.02	0.248	1.80	0.67–4.81	0.244	0.72	0.10–5.13	0.741
NMSC	3267	**0.67**	**0.48–0.95**	**0.025**	0.74	0.45–1.23	0.245	**0.34**	**0.13–0.93**	**0.035**
Prostate	1461	0.73	0.47–1.14	0.164	0.65	0.31–1.38	0.263	0.87	0.41–1.85	0.710
Non-smoking										
Breast	3682	0.83	0.56–1.22	0.339	0.68	0.37–1.23	0.202	0.93	0.38–2.31	0.884
Colon	459	1.03	0.49–2.18	0.930	1.03	0.33–3.19	0.965	**3.64**	**1.49–8.88**	**0.004**
Melanoma of skin	627	0.78	0.32–1.87	0.572	1.02	0.33–3.16	0.978	0.80	0.11–5.68	0.819
NMSC	3577	0.89	0.66–1.22	0.481	0.80	0.50–1.29	0.365	0.80	0.39–1.62	0.527
Prostate	1233	0.63	0.36–1.09	0.096	0.43	0.16–1.17	0.097	-	-	-
Alcohol										
Breast	6309	1.09	0.83–1.42	0.529	0.92	0.61–1.38	0.682	1.16	0.63–2.15	0.627
Colon	984	0.83	0.46–1.50	0.536	0.71	0.27–1.90	0.494	1.60	0.66–3.88	0.293
Melanoma of skin	1023	1.18	0.68–2.04	0.550	1.41	0.67–2.98	0.361	0.82	0.20–3.28	0.776
NMSC	6618	0.81	0.64–1.02	0.070	0.79	0.56–1.12	0.189	**0.51**	**0.27–0.95**	**0.034**
Prostate	2639	**0.70**	**0.50–1.00**	**0.051**	0.59	0.32–1.07	0.082	0.58	0.27–1.22	0.149
Non-alcohol										
Breast	362	0.29	0.07–1.18	0.084	0.62	0.14–2.67	0.523	-	-	-
Colon	37	-	-	-	-	-	-	-	-	-
Melanoma of skin	46	-	-	-	-	-	-	-	-	-
NMSC	240	0.43	0.11–1.77	0.244	0.60	0.08–4.38	0.615	1.81	0.42–7.90	0.429
Prostate	71	0.50	0.07–3.74	0.502	-	-	-	-	-	-

Bold text indicates a *p*-value less than 0.05, which is statistically significant

− The number of people diagnosed with ACD, AD, or VD in the types of cancer is too small to calculate the HR

*Abbreviations*: *ACD* all-cause dementia, *AD* Alzheimer’s disease, *VD* vascular dementia, *HR* hazard ratios, *CI* confidence interval, *NMSC* non-melanoma skin cancer

^a^Common cancer and more than 1000 people with cancer

^b^Calculated using Cox proportional hazards modeling, adjusted for age, sex, education, *ApoE4*, socioeconomic status, BMI, smoking status, alcohol consumption, diabetes, hypertension, heart failure, obesity, and stroke

## Discussion

### Summary of results

In this nationwide prospective cohort study, our results showed that cancer was associated with modestly reduced risks of ACD and VD after adjusting for common dementia risk factors. Subgroup analyses showed that the association of cancer with subsequent dementia risk was more pronounced in older people, males, non-carriers of *ApoE4*, and those with higher BMI. In addition, we observed that reproductive system cancer was associated with a lower risk of ACD, whereas male genital system cancer had strong inverse associations with both ACD and AD risks. Then NMSC and PC were associated with a lower risk of ACD.

### Comparison with previous literature

Several studies have investigated the relationship between cancer and dementia especially AD, yielding inconsistent findings. Some reported inverse associations [18–20], while others revealed positive [30, 31] or null connections [7, 28]. Our results supported that cancer was related to a lower risk of dementia was consistent with a review of previous experimental studies, prospective cohort studies, and meta-analyses [32]. Similar to the results of Ording et al. and Sun et al. [12, 20], the current study found a stronger association between cancer and the risk of ACD and VD compared to AD after further adjustment for other potentially confounding factors besides age and sex. Similar to our results, a recent prospective cohort study showed that cancer was associated with a roughly 42% reduction in risk of dementia and a 55% reduction in risk of AD [18]. In addition, a study from the Utah Population Database argued that the inverse association between cancer and AD risk arose from bias due to the competing risks of death [17]. However, this association remained stable after controlling for competing risks of death in this study. Similarly, one recent study also suggested that the bias induced by selective survival in simulations was too small to explain the observed inverse cancer-dementia link [33]. Previous studies showed that medical treatments for the cancer would cause a decline in the memory [34, 35]. However, we found an inverse association between cancer and ACD risk after adjusting for cognitive function. The effect of cognitive function on the relationship between cancer and dementia risk was not found in the present study.

Driver et al. found that patients with smoking-related cancers had a lower risk of probable AD than those with non-smoking-related cancer [11], but there were no correlations between smoking-related cancers and dementia risk in our study. This inconsistency might be due to our adjustment for additional confounders. Consistent with the study of Ording et al. and we had found that cancer was found to be protective against dementia when individuals aged over 60 years old. This may be explained by the fact that aging is a common risk factor for both dementia [36] and cancer [37]. Compared with previous studies, the present study included more subgroup analyses of cancer types and systems to further explore the correlation between cancer and dementia risk. Our findings showed that patients of male genital system cancer had lower risks of dementia and AD because of the inverse association between prostate cancer and dementia. Previous related studies produced inconsistent results, possibly due to the differences in sample size between studies [20, 31]. A recent cohort study reported the inverse association between NMSC and dementia is stronger for ACD than for other dementia types [38], which was consistent with the current study. However, we found no significant association between other cancers and AD after excluding NMSC, which was inconsistent with Driver et al. [11]. This may be due to their smaller sample size and correction factors.

Several studies investigated whether cancer treatment modulates the risk of dementia. Cancer treatment might be associated with a cancer diagnosis, and also might engender temporary effects on cognition like “chemobrain” [39]. Although chemotherapy might impair cognitive function, there is no clear evidence linking chemotherapy with an increased risk of dementia. Baxter et al. [40] and Du et al. [41] found patients receiving chemotherapy had a reduced dementia risk, which might be explained that chemotherapy can protect neurons susceptible to AD by inhibiting inflammation [42]. Recent evidence found that anti-cancer drugs targeting mTOR slow AD progression by reducing iron accumulation, suggesting that selective anticancer drugs might have a modulating effect on AD pathophysiology [43]. Recent meta-analyses showed that individuals with prostate cancer who received androgen deprivation therapy (ADT) had an increased risk of subsequent dementia [44, 45]. This was contrary to our findings on prostate cancer on dementia risk. Therefore, whether or not to receive cancer treatment might have an impact on the risk of dementia after a cancer diagnosis.

### Potential mechanisms

Although cancer patients might have some protection from dementia, the underlying pathophysiological mechanism was not yet clearly defined. The inverse association between cancer and dementia might be related to differential regulation of common genes and pathways. In cancer, cell regulation mechanisms were disrupted with increased cell survival and/or proliferation, whereas AD, in contrast, was associated with increased neuronal death caused by or accompanied by deposition of amyloid-beta (Aβ) and tau. Gene polymorphisms, DNA methylation, or other mechanisms that induce changes in molecular activity play critical roles in the decision to “repair and live”- or “die” and may be involved in the pathogenesis of both diseases [13]. For example, a recent review proposes that several molecular players, namely p53 and PIN1, may be involved in complex molecular interaction associated with this inverse correlation [37]. The prototypical tumor suppressor protein p53 plays opposite roles in the pathological development of cancer and dementia due to the abnormal activation of other proteins [37]. As seen in polymorphisms of p53, a genetic predisposition to anti-apoptosis might protect individuals from cancer while increasing the risk of neurodegeneration [46]. The protein Pin1 is a ubiquitous enzyme that has been shown to regulate a diverse array of cellular processes of cell proliferation and differentiation [47]. Many tumors such as prostate and lung cancer in humans over-express Pin1 [48], whereas its expression in AD patient’s brain is very low [37]. Single nucleotide polymorphisms (SNPs) in the promoter region of the Pin1 gene that inhibit Pin1 expression are associated with increased AD risk [49] and decreased cancer risk [50]. Taken together, these findings provide evidence for a true protective role of cancer in reducing dementia risk.

## Strengths and limitations

There were several strengths and limitations of this population cohort study. Major strengths of the current study were its large sample size of UK Biobank participants, the prospective design, and long-term follow-up. All cancer and dementia patients were identified from the nationwide databases with high accuracy and coverage. The use of nationally registered data can guarantee the integrity of the data. In addition, the UK Biobank cohort collected extensive information on behaviors, sociodemographic risk factors, and disease history, allowing adjustment for potential confounders associated with dementia. These approaches limited potential problems with selection bias, sample size, and generalizability. Due to the large sample size, we were able to investigate potential differential effects on dementia risk according to cancer sites and systems. Moreover, we adjusted for various dementia risk factors that may confound the association of cancer with dementia.

This study also had some limitations. First, dementia diagnoses were obtained from registry-based data rather than detailed neuropsychological assessments. Although the overall accuracy of obtaining dementia diagnoses through registries is good [24], there is still the potential to misclassify some study participants. Furthermore, although there is evidence that the rate of false positives is relatively low, the rate of false negatives is still largely unknown [24]. Second, the number of dementia cases was small for some rare cancer types. Third, the relatively young age of the cohort limited the number of cases of dementia, especially AD, thus limiting our ability to identify correlations. Forth, we lacked information on the cancer treatment history of the participants. During the follow-up period, cancer survivorship and dementia diagnosis rate showed differences between participants with and without cancer treatment. Finally, the present analyses were performed only within individuals of white British descent and hence findings might not be generalizable to other ethnicities and general populations. It cannot be used to provide representative disease prevalence and incidence rates. Nevertheless, an effective assessment of exposure-disease correlations is generalizable and does not require participants to be representative of the entire population [51].

## Conclusions

In conclusion, the findings from this large cohort study were consistent with previous studies that cancer was associated with a reduction in the risk of dementia. This study showed that NMSC and PC were associated with reduced dementia risk. Future research is required to further explore the mechanistic basis of this relationship to improve understanding of the mechanisms underlying this negative association. In addition, investigating the potential impact of cancer treatment on dementia risk is another interesting area for future work.

## Supplementary Information

**Additional file 1: Supplementary Figure 1.** Kaplan-Meier analysis for the cumulative incidence of dementia with log-rank test. **Supplementary Figure 2.** The survival curves of cumulative incidence of dementia and competing risk event for death. **Supplementary Table 1.** Association of cancer with dementia risk after additional adjustment for cognitive function. **Supplementary Table 2.** Association of site-specific cancers with dementia and its subtypes. **Supplementary Table 3.** Association of specific cancer sites with ACD, AD, and VD by *ApoE4* status and BMI.

## Data Availability

The datasets described in this manuscript are available from the UK Biobank with an approved protocol under the project number of 19,542. External investigators can request the data and approval of use on application to the UK Biobank (www.ukbiobank.ac.uk/).

## Abbreviations

HRs: Hazard ratios
CIs: Confidence intervals
ACD: All cause dementia
AD: Alzheimer’s disease
VD: Vascular dementia
The UK: The United Kingdom
ICD: The International Statistical Classification of Diseases
PPV: Positive predictive value
ApoE4: Apolipoprotein E ε4
BMI: Body mass index
SD: Standard deviation
NMSC: Non-melanoma skin cancer
IQR: Interquartile range
PC: Prostate cancer
ADT: Androgen deprivation therapy
Aβ: Amyloid-beta
SNPs: Single nucleotide polymorphisms
