# Supplementary material for: Investigating the association between cancer and dementia risk: a longitudinal cohort study
Source: Alzheimers Res Ther. 2022 Oct 5;14:146. doi: 10.1186/s13195-022-01090-9 (PMC9533604; doi:10.1186/s13195-022-01090-9)
Supplement: Supplementary file 1 — Additional file 1: Supplementary Figure 1. Kaplan-Meier analysis for the cumulative incidence of dementia with log-rank test. Supplementary Figure 2. The survival curves of cumulative incidence of dementia and competing risk event for death. Supplementary Table 1. Association of cancer with dementia risk after additional adjustment for cognitive function. Supplementary Table 2. Association of site-specific cancers with dementia and its subtypes. Supplementary Table 3. Association of specific cancer sites with ACD, AD, and VD by ApoE4 status and BMI. [file 13195_2022_1090_MOESM1_ESM.docx]

**Supplementary materials**

**Supplementary Figure 1 Kaplan-Meier analysis for the cumulative incidence of dementia with log-rank test**

1. All cause dementia B. AD C. VD


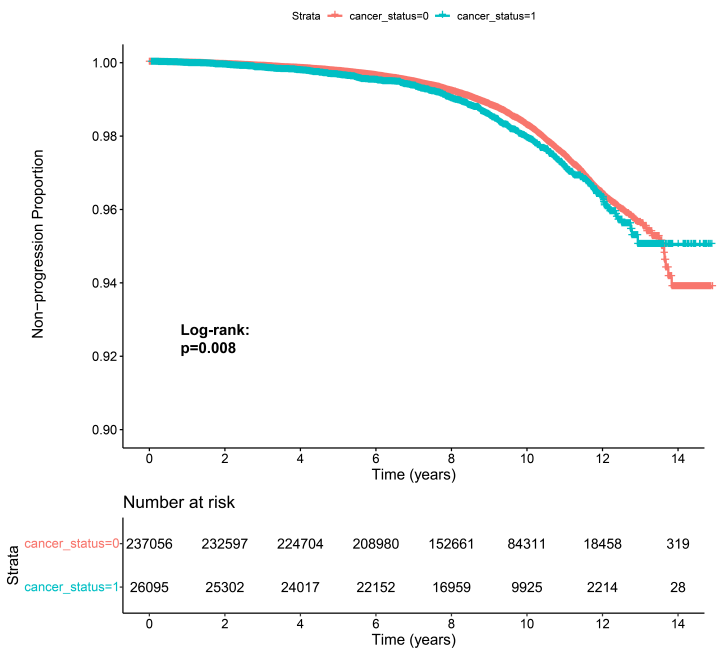

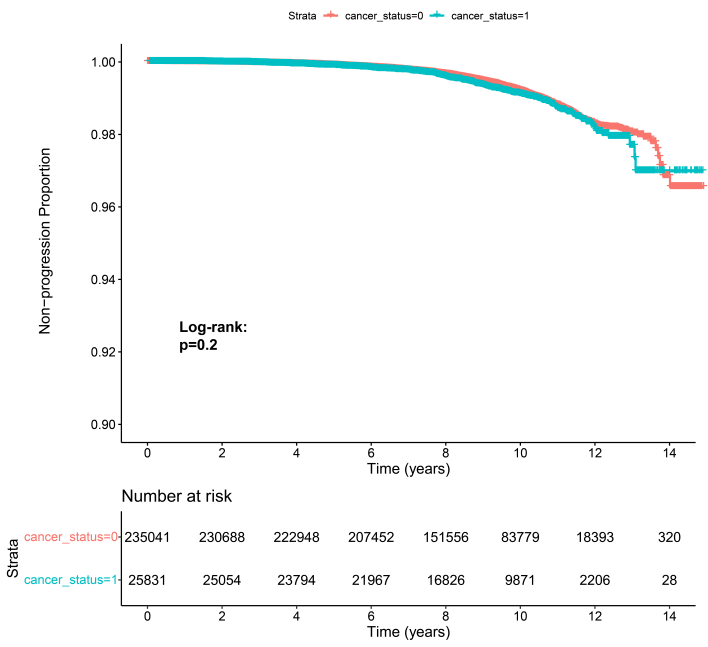

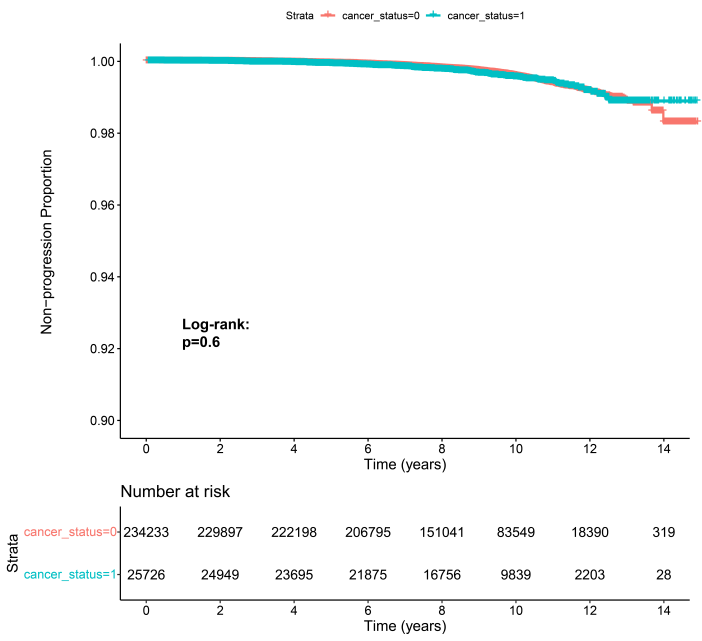


Abbreviation: AD, Alzheimer’s disease; VD, vascular dementia.

**Supplementary Figure 2 The survival curves of cumulative incidence of dementia and competing risk event for death**

1. All cause dementia B. AD C. VD


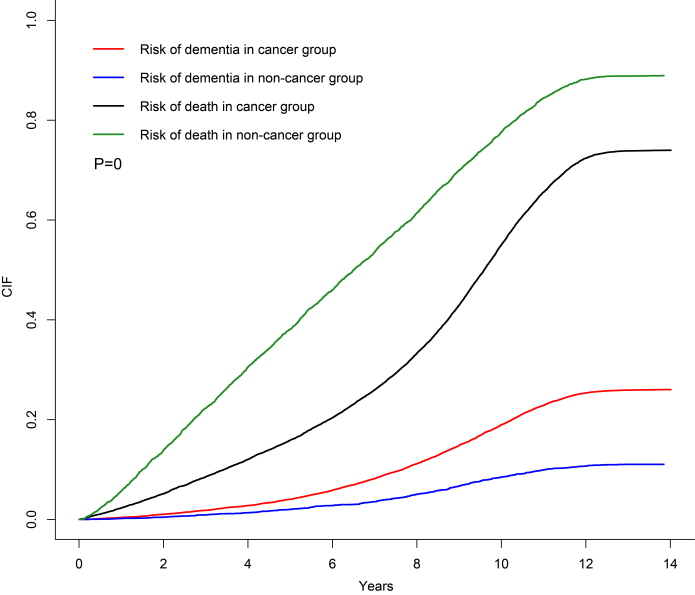

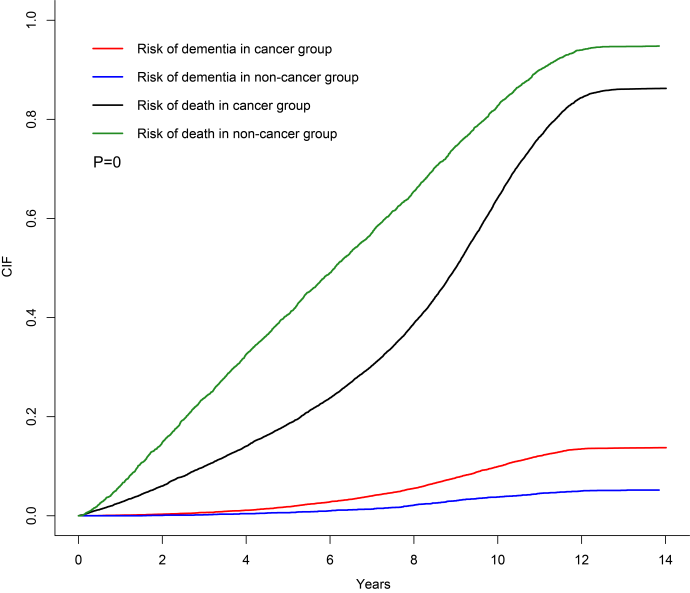

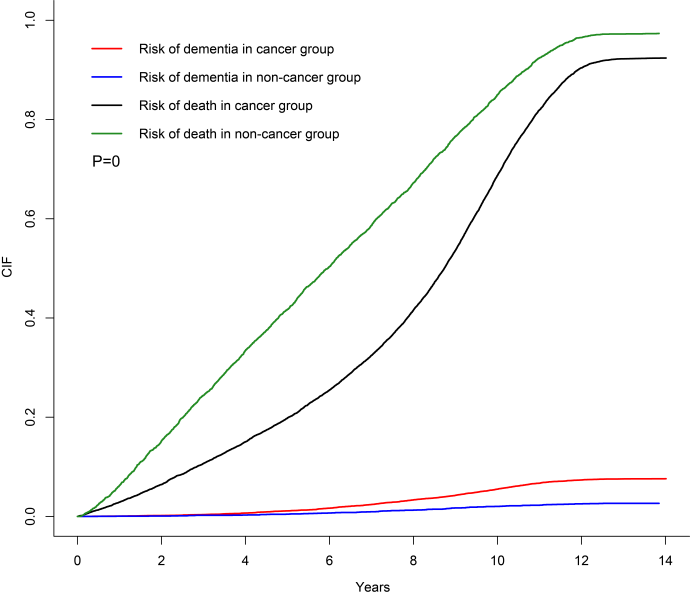


Abbreviation: CIF: cumulative incidence probability; AD, Alzheimer’s disease; VD, vascular dementia.

**Supplementary Table 1 Association of cancer with dementia risk after additional adjustment for cognitive function**

| Type of dementia | HR* | 95%CI | *P*-value |
| --- | --- | --- | --- |
| ACD | **0.86** | **0.76-0.98** | **0.024** |
| AD | 0.87 | 0.72-1.05 | 0.157 |
| VD | 0.75 | 0.57-1.01 | 0.055 |

Abbreviation: ACD, all cause dementia; AD, Alzheimer’s disease; VD, vascular dementia; HR, hazard ratios; CI, confidence interval.

*Calculated using Cox proportional hazards modelling, adjusted for age, sex, education, APOE4, socioeconomic status, BMI, smoking status, alcohol consumption, diabetes, hypertension, heart failure, obesity, stroke and cognitive function (pairs matching and reacting time).

**Supplementary Table 2 Association of site-specific cancers with dementia and its subtypes**

| Cancer sites# | Participants with cancer history (N) | All cause dementia | | | AD | | | VD | | |
| --- | --- | --- | --- | --- | --- | --- | --- | --- | --- | --- |
|  |  | HR* | 95%CI | *P*-value | HR* | 95%CI | *P*-value | HR* | 95%CI | *P*-value |
| Non-cancer | 237,056 | Reference |  |  | Reference |  |  | Reference |  |  |
| Any cancer (except NMSC) | 19,231 | 0.90 | 0.78-1.04 | 0.163 | 0.90 | 0.72-1.12 | 0.331 | 0.81 | 0.58-1.12 | 0.197 |
| Smoking related cancer | 1,689 | 0.72 | 0.45-1.17 | 0.186 | 1.30 | 0.75-2.26 | 0.345 | 0.60 | 0.22-1.60 | 0.303 |
| Non-smoking related cancer | 24,406 | 0.88 | 0.77-1.00 | 0.053 | 0.83 | 0.68-1.01 | 0.068 | 0.75 | 0.56-1.01 | 0.059 |
| Bladder | 587 | 0.79 | 0.41-1.53 | 0.487 | 1.67 | 0.83-3.35 | 0.153 | 0.87 | 0.28-2.71 | 0.809 |
| Breast | 6,682 | 1.01 | 0.78-1.32 | 0.922 | 0.90 | 0.61-1.34 | 0.612 | 1.04 | 0.56-1.91 | 0.903 |
| Bronchus or lung | 210 | 1.02 | 0.26-4.10 | 0.973 | 1.21 | 0.17-8.59 | 0.851 | 1.81 | 0.25-12.94 | 0.552 |
| Cervix uteri | 145 | 0.96 | 0.14-6.85 | 0.970 | 1.98 | 0.28-14.07 | 0.496 | - | - | - |
| Colon | 1,021 | 0.81 | 0.45-1.46 | 0.474 | 0.69 | 0.26-1.85 | 0.463 | 1.58 | 0.65-3.82 | 0.310 |
| Corpus uteri | 610 | 1.32 | 0.66-2.65 | 0.436 | 1.31 | 0.49-3.50 | 0.592 | - | - | - |
| Kidney | 347 | 0.51 | 0.13-2.06 | 0.346 | 0.61 | 0.09-4.33 | 0.620 | - | - | - |
| Larynx | 128 | 0.71 | 0.10-5.02 | 0.728 | 1.86 | 0.26-13.23 | 0.536 | - | - | - |
| Leukemia | 322 | 0.74 | 0.24-2.31 | 0.607 | - | - | - | 1.09 | 0.15-7.74 | 0.934 |
| Lymphoma | 614 | 0.85 | 0.40-1.78 | 0.660 | 1.16 | 0.43-4.10 | 0.765 | - | - | - |
| Melanoma of skin | 1,069 | 1.11 | 0.64-1.91 | 0.718 | 1.34 | 0.64-2.81 | 0.445 | 0.78 | 0.19-3.13 | 0.726 |
| Multiple myeloma | 135 | 0.64 | 0.09-4.52 | 0.651 | 1.37 | 0.19-9.77 | 0.751 | 2.26 | 0.32-16.08 | 0.418 |
| **NMSC** | 6,864 | **0.79** | **0.62-0.99** | **0.042** | 0.78 | 0.56-1.10 | 0.162 | 0.57 | 0.32-1.02 | 0.057 |
| Oesophagus or stomach | 206 | 0.65 | 0.16-2.62 | 0.550 | 0.81 | 0.11-5.77 | 0.833 | - | - | - |
| Ovary | 364 | 0.36 | 0.05-2.52 | 0.301 | 0.69 | 0.10-4.88 | 0.706 | - | - | - |
| **Prostate** | 2,712 | **0.69** | **0.49-0.97** | **0.035** | 0.56 | 0.31-1.02 | 0.058 | 0.54 | 0.25-1.14 | 0.106 |
| Rectum | 595 | 0.91 | 0.41-2.04 | 0.828 | 0.36 | 0.05-2.59 | 0.313 | 1.33 | 0.33-5.34 | 0.688 |
| Thyroid | 169 | 2.84 | 0.91-8.82 | 0.071 | 3.69 | 0.92-14.82 | 0.065 | - | - | - |

Abbreviation: AD, Alzheimer’s disease; VD, vascular dementia; HR, hazard ratios; CI, confidence interval; NMSC, non-melanoma skin cancer.

# Specific cancer sites only included those with cancers in at least 100 affected individuals and with at least 10 dementia cases among the affected individuals.

Smoking related cancer: oral, pharynx, larynx, oesophagus, stomach, pancreas, bronchus or lung, cervix uteri, bladder, and kidney.

*Calculated using Cox proportional hazards modelling, adjusted for age, sex, education, APOE4, socioeconomic status, BMI, smoking status, alcohol consumption, diabetes, hypertension, heart failure, obesity, and stroke.

Bold text indicates a p-value less than 0.05, which is statistically significant.

-The number of people diagnosed with dementia, AD, or VD in the type of cancer patients is too small to calculate the HR value.

**Supplementary Table 3 Association of specific cancer sites with ACD, AD, and VD by APOE4 status and BMI**

| Specific cancer sites^ | Participants with cancer history (N) | All cause dementia | | | AD | | | VD | | |
| --- | --- | --- | --- | --- | --- | --- | --- | --- | --- | --- |
|  |  | HR* | 95% CI | *P*-value | HR* | 95% CI | *P*-value | HR* | 95% CI | *P*-value |
| APOE 4 carrier |  |  |  |  |  |  |  |  |  |  |
| Breast | 1,762 | 1.16 | 0.84-1.60 | 0.382 | 0.99 | 0.62-1.57 | 0.965 | 1.34 | 0.62-2.89 | 0.459 |
| Colon | 295 | 0.57 | 0.21-1.52 | 0.263 | 0.56 | 0.14-2.24 | 0.413 | 2.31 | 0.74-7.26 | 0.150 |
| Melanoma of skin | 292 | 1.20 | 0.57-2.52 | 0.634 | 1.53 | 0.63-3.70 | 0.343 | 0.96 | 0.14-6.88 | 0.971 |
| NMSC | 1,883 | 0.85 | 0.63-1.15 | 0.284 | 0.92 | 0.62-1.37 | 0.678 | 0.94 | 0.50-1.78 | 0.858 |
| Prostate | 704 | 0.62 | 0.37-1.04 | 0.070 | 0.61 | 0.29-1.29 | 0.193 | 0.32 | 0.08-1.29 | 0.109 |
| APOE 4 non-carrier |  |  |  |  |  |  |  |  |  |  |
| Breast | 4,444 | 0.82 | 0.52-1.29 | 0.392 | 0.73 | 0.34-1.55 | 0.411 | 0.76 | 0.28-2.08 | 0.595 |
| Colon | 688 | 1.04 | 0.49-2.18 | 0.926 | 0.86 | 0.21-3.46 | 0.834 | 1.07 | 0.27-4.33 | 0.920 |
| Melanoma of skin | 746 | 0.97 | 0.43-2.16 | 0.935 | 0.93 | 0.23-3.72 | 0.913 | 0.65 | 0.09-4.68 | 0.673 |
| **NMSC** | 4,782 | 0.73 | 0.51-1.05 | 0.090 | 0.58 | 0.30-1.13 | 0.107 | **0.20** | **0.05-0.79** | **0.022** |
| Prostate | 1,935 | 0.76 | 0.48-1.22 | 0.254 | 0.50 | 0.18-1.34 | 0.166 | 0.74 | 0.30-1.81 | 0.513 |
| 18≤BMI<25 |  |  |  |  |  |  |  |  |  |  |
| Breast | 2,308 | 0.93 | 0.60-1.43 | 0.738 | 0.81 | 0.43-1.52 | 0.508 | 1.10 | 0.40-3.05 | 0.853 |
| Colon | 282 | 0.86 | 0.32-2.30 | 0.763 | 0.86 | 0.21-3.47 | 0.835 | 1.86 | 0.46-7.53 | 0.384 |
| **Melanoma of skin** | 341 | 1.92 | 0.86-4.29 | 0.113 | **2.77** | **1.03-7.45** | **0.043** | **4.48** | **1.10-18.32** | **0.037** |
| NMSC | 2,306 | 1.03 | 0.74-1.45 | 0.849 | 1.06 | 0.66-1.70 | 0.811 | 0.89 | 0.39-2.01 | 0.774 |
| Prostate | 630 | 1.20 | 0.73-1.95 | 0.472 | 0.84 | 0.34-2.05 | 0.701 | 0.77 | 0.24-2.46 | 0.657 |
| 25≤BMI<30 |  |  |  |  |  |  |  |  |  |  |
| Breast | 2,669 | 1.29 | 0.87-1.91 | 0.200 | 1.03 | 0.56-1.89 | 0.928 | 1.50 | 0.60-3.77 | 0.387 |
| Colon | 458 | 0.69 | 0.26-1.86 | 0.467 | 0.42 | 0.06-3.02 | 0.392 | 0.71 | 0.10-5.09 | 0.734 |
| Melanoma of skin | 468 | 0.78 | 0.32-1.88 | 0.581 | 0.34 | 0.05-2.44 | 0.285 | - | - | - |
| **NMSC** | 3,040 | **0.66** | **0.45-0.97** | **0.032** | 0.63 | 0.36-1.12 | 0.117 | 0.66 | 0.29-1.50 | 0.326 |
| Prostate | 1,378 | 0.50 | 0.28-0.92 | 0.025 | 0.45 | 0.17-1.21 | 0.114 | 0.68 | 0.25-1.85 | 0.450 |
| BMI≥30 |  |  |  |  |  |  |  |  |  |  |
| Breast | 1,659 | 0.77 | 0.41-1.46 | 0.426 | 0.90 | 0.36-2.22 | 0.817 | 0.63 | 0.15-2.58 | 0.521 |
| Colon | 275 | 0.95 | 0.30-2.95 | 0.928 | 0.89 | 0.12-6.34 | 0.906 | 2.54 | 0.63-10.29 | 0.192 |
| Melanoma of skin | 260 | 1.07 | 0.27-4.31 | 0.921 | 2.86 | 0.71-11.54 | 0.141 | - | - | - |
| NMSC | 1,470 | 0.66 | 0.36-1.19 | 0.167 | 0.60 | 0.22-1.62 | 0.313 | - | - | - |
| Prostate | 692 | 0.44 | 0.18-1.08 | 0.072 | 0.44 | 0.11-1.78 | 0.248 | - | - | - |

Abbreviation: ACD, all cause dementia; AD, Alzheimer’s disease; VD, vascular dementia; APOE 4, apolipoprotein E ε4; BMI, body mass index; HR, hazard ratios; CI, confidence interval; NMSC, non-melanoma skin cancer.

^ Common cancer and more than 1000 people with cancer.

*Calculated using Cox proportional hazards modelling, adjusted for age, sex, education, APOE4, socioeconomic status, BMI, smoking status, alcohol consumption, diabetes, hypertension, heart failure, obesity, and stroke.

**-**The number of people diagnosed with dementia, AD, or VD in the type of cancer patients is too small to calculate the HR value.
